# Learned Shrinkage Approach for Low-Dose Reconstruction in Computed Tomography

**DOI:** 10.1155/2013/609274

**Published:** 2013-06-20

**Authors:** Joseph Shtok, Michael Elad, Michael Zibulevsky

**Affiliations:** Computer Science Department, Technion - Israel Institute of Technology, Haifa 32000, Israel

## Abstract

We propose a direct nonlinear reconstruction algorithm for Computed Tomography (CT), designed to handle low-dose measurements. It involves the filtered back-projection and adaptive nonlinear filtering in both the projection and the image domains. The filter is an extension of the learned shrinkage method by Hel-Or and Shaked to the case of indirect observations. The shrinkage functions are learned using a training set of reference CT images. The optimization is performed with respect to an error functional in the image domain that combines the mean square error with a gradient-based penalty, promoting image sharpness. Our numerical simulations indicate that the proposed algorithm can manage well with noisy measurements, allowing a dose reduction by a factor of 4, while reducing noise and streak artifacts in the FBP reconstruction, comparable to the performance of a statistically based iterative algorithm.

## 1. Introduction

### 1.1. Problem Statement

 Computed tomography (CT) imaging produces a 3D map of the scanned object, where the different materials are distinguished by their X-ray attenuation properties. In medicine, such a map has a great diagnostic value, making the CT scan one of the most frequent noninvasive exploration procedures practiced in almost every hospital. The attenuation of biological tissues is measured by comparing the intensity of the X-rays entering and leaving the body. The main problem precluding pervasive use of the CT scan for diagnostics and monitoring is the damage caused to the tissues by the X-ray radiation. CT manufacturers make great efforts to reduce the X-ray dose required for images of diagnostic quality. In this work we propose an algorithm that enables a high-quality reconstruction from low-dose (and thus noisy) measurements.

In ideal conditions, the information obtained in the scan suffices to build an exact attenuation map, called the CT image. In practice, the measurements are degraded by a number of physical phenomena. The main factors are off-focal radiation, afterglow and crosstalk in the detectors, beam hardening, and Compton scattering (see [1] for a detailed overview). These introduce a structured error into the measurements, mostly the type that is modeled by a convolution with some kernel. Another source of deterioration, dominant in the low-dose scenario, is the stochastic noise. One type of such noise stems from the low photon counts, which occur when the X-rays pass through high-attenuation areas. This phenomenon is similar to the shot noise, encountered in photo cameras in poor lighting conditions. Statistically, the photon counts are modeled as instances of Poisson random variables. Another type of the stochastic noise originates from dark currents in the detectors, interference noise from interconnecting cables, and other hardware sources. This electronic noise is modeled in the measurements as an additive Gaussian random variable.

In this work we aim to reduce the influence of the stochastic noise on the image quality, with the assumption that the structured error components, mentioned above, are treated by the existing methods. Explicitly, we use the well-accepted compound Poisson-Gaussian statistical model and propose a new noniterative method for image reconstruction, based on the concept of sparse representations [2] and involving machine learning concepts.

### 1.2. Present Reconstruction Algorithms

 The basic linear reconstruction method, filtered back projection (FBP) [3], makes a very limited account of the noise structure in the data: it employs a low-pass 1D convolution filter in the Radon domain, whose parameters are preset for specific anatomical regions and standard scan protocols. Errors in the photon counts manifest in the output CT image in the form of streak artifacts, which corrupt its content and jeopardize its diagnostic value. Accordingly, each measured line integral is effectively smeared back over that line by the back projection; an incorrect measurement results in a line of wrong intensity in the image. Typically, the streaks radiate from bone regions or metal implants, which corrupt its content and jeopardize its diagnostic value. Images of better quality—with reduced artifacts and increased spatial resolution—are obtained with a statistically-based approach, where the maximum a posteriori (MAP) is optimized with respect to the sought image. This problem is converted to a minimization of the penalized likelihood (PL) objective function [1], (1). The likelihood expression models the physical process of CT scan, which allows interpreting the measurements more correctly. The likelihood component expresses the expected statistical behavior of the data. The penalty component models the expected properties of the CT images (i.e., contains a *prior* information about the image to be reconstructed). The PL objective can be designed to restore the measurements from noisy observations [1, 4, 5] or to reconstruct directly the CT image [6]. In most cases, the optimization problem is difficult to solve, so it is sometimes replaced by a second-order approximation, the penalized weighted least squares (PWLS) [6, 7]. A drawback of a reconstruction based on explicit statistical modeling is the computationally heavy iterative solution.

To improve the performance of the fast FBP reconstruction, adaptive signal processing techniques, implicitly modeling the noise statistics, have been proposed. These are applied to the measurements data in a noniterative fashion and have computational complexity comparable to that of the FBP. Hsieh employs a trimmed mean filter, adaptive to the noise variance [8]. For each detector reading *x*, the algorithm adaptively chooses a number of its neighbors participating in the filtering operation. The value of *x* is replaced with the average of these neighbors after a portion of their highest and lowest values is discarded. To some extent, the aforementioned statistical model of the scan is used: the noisy samples get a stronger filtering than the more reliable ones. The experimental results in this work are very impressive. A similar concept, with a different kind of filter, is adopted by the work of Kachelrieß et al. who apply adaptive convolution-based filtering in the sinogram domain [9]. The filter width is data dependent and also it is applied only where the data intensity is below a threshold, so the algorithm processes only the regions where the noise is substantial. In [10], two signal processing steps are introduced to improve the performance of FBP. The measurements' data is processed by the penalized weighted least squares filter in a Karhunen-Loeve domain (more familiar under the name of principal component analysis, PCA). An additional step of image postprocessing is performed by edge-preserving smoothing with locally adaptive parameters.

Beyond the use of general-purpose tools, there are algorithms applying machine learning methods for adaptive processing of the tomographic data. Close in its spirit to our work is the algorithm described in [11]. Here, the measured projections are locally filtered according to a preliminary classification of its regions. The classes and the corresponding filters are derived automatically, via an offline example-based training process. Thus, the standard smoothing by the low-pass convolution filter is replaced with locally adaptive filtering, optimized for the minimal mean square error in the training images.

### 1.3. Our Work

 Our method employs an adaptive local processing of the measurements and a matched postprocessing stage in the CT image domain. Those steps are intended to enable the FBP to deal with the noisy measurements. The technique employed in both stages is a learned shrinkage in the transform domain, following the ideas outlined in the work by Hel-Or and Shaked in [12].

The learned shrinkage filter was originally designed for noise reduction and is employed in our work to reduce an error measure in the CT image domain, while acting on the raw measurements (at the first stage) and on the reconstructed image (at the second). In a nutshell, the learned shrinkage operator is a nonlinear adaptive filter, applied locally to the signal data. It requires an example-based training of the filter's defining parameters, which minimizes a desired reconstruction error with respect to reference CT images.

Due to the fact that the noise in the measurements is data dependent, we introduce a scalar transformation which normalizes the noise variance according to its statistical model. The aforementioned filter is applied after this transformation. The error measure in the image domain, used in the training objective, is a function of the reconstructed CT image and its ground truth—a high quality reference image. The measure consists of two components, the standard mean square error and a gradient-based expression capturing the amount of blur at fine edges of the image. Filters, optimized with respect to this error measure, are shown to produce CT images with low spatial noise and artifacts, while producing sharp edges.

On one hand, our approach accounts for the statistical model of the noise, as used by the iterative algorithms; on the other, the computationally heavy learning procedure is performed once offline, at the calibration stage, while the processing of the new data is done very fast, on par with the FBP algorithm. This gives a hope to bridge the gap between the slow high-quality statistical algorithms and the fast linear reconstruction. We also mention that in the learning process our algorithm has the potential to adjust to additional unknown degradation factors and hardware specifications.

One possible application of the proposed method is to exploit the adaptive nature of its filtering stages to taylor the filter parameters to an individual patient, in a specific scan setup. Such step can be made in the scenario where repeated CT scans are performed, for reasons of monitoring. Thus after the first full-dose scan, the X-ray exposure in the following procedures can be reduced by using filters trained on the image data very similar to that which is expected in the next scan. With the correct training protocol, there is no danger of overfitting the filters to the “healthy” images and thus to jeopardise detection of anomalies (an experiment, suggesting this fact, is reported later in this paper). In this way, a patient can avoid a substantial amount of X-ray exposure.

In our numerical experiments we compare the proposed algorithm against three existing ones. First is the optimally tuned FBP, which serves as a baseline; another is the nonlinear ATM filter for CT reconstruction, proposed by Hsieh in [8], and the third is the iterative, statistically-based PWLS reconstruction [6]. Our method is shown to outperform both the FBP and the ATM in the sense of image quality and robustness to changes of the anatomical region, and it is comparable to the statistically-based reconstruction. The comparison includes a visual display, a number of quantitative measures and evaluation of the local impulse response for each algorithm.

The paper is organized as follows. The mathematical description of the CT scan is given in Section 2. The learned shrinkage method is presented in Section 3. The new error measure is described in Section 4, laying the ground for our method for CT reconstruction, described in Section 5. A numerical study is given in Section 6.  Section 7 concludes the paper.

## 2. Mathematical Model of the CT Scan

 Our algorithm is designed in the setup of two-dimensional, parallel-beam scan geometry. An example of a scanned object is an axial slice (axial plane is the one parallel to the floor when the patient is standing) of the patient's body. The main part of a CT scanner is a rotating gantry, which has the X-ray source mounted against an array of detectors. During the scan, the gantry sweeps the angular range of [0, *π*], equally divided into a large number of projections or “views”. For each angle *θ*, the one-dimensional array of detectors produces photon counts from rays that arrive in a fan-shaped beam from the source. Via a rebinning step, the rays are rearranged so that there is a comb of parallel rays for each projection. The acquired data is arranged into a 2D matrix, whose columns correspond to different angles, and the rows are assigned to different bins in each projection.

To describe the nature of measured data we use the compound Poisson-Gaussian statistical model that is assumed in [1, 13] and is also empirically verified in [8]. Each measured photon count *y*_*ℓ*_ is viewed as an instance of the random variable *Y*_*ℓ*_ given by

(1) Yℓ~Poiss(λℓ)+Gauss(d,σn), whereλℓ=λ0·exp⁡(−[Rf]ℓ),

where **R** *f* is the Radon transform of the scanned image *f* and the constant *λ*_0_ is the photon count at the X-ray source. The Radon transform is defined on the collection of all straight lines *ℓ* through the object. For each *ℓ*, its value is the linear integral:

(2) $$\begin{array}{c} {\left[Rf\right]}_{\ell }=\int_{\ell }f\left(\ell \right)d\ell . \end{array}$$

The log-transformed photon counts *g*_*ℓ*_ = −log⁡(*y*_*ℓ*_/*λ*_0_) are the approximate line integrals; the corresponding data matrix *g* is called a *sinogram* since every point in the image space traces a sine curve in this domain.

The filtered back-projection algorithm is based on the Radon inversion formula. First, the measurements are transformed to the Radon domain by the logarithm function: *g* = −log⁡(*y*/*λ*_0_). Then, a linear operator implementing the inverse of Radon transform is applied as follows:

(3) $$\begin{array}{c} \tilde{f}=R^{*}F_{\text{RL}}\left(g\right), \end{array}$$

where R* is the adjoint of the Radon transform, also known as the back-projection operator:

(4) $$\begin{array}{c} \left(R^{*}g\right)x=\int_{\theta }g\left(\theta ,x\cdot \theta \right)d\theta . \end{array}$$

The filter **F**_RL_ uses the Ram-Lak kernel *κ* [14], defined in the Fourier domain by

(5) $$\begin{array}{c} \hat{\kappa }\left(\omega \right)=\left|\omega \right|. \end{array}$$

In practice, an additional, low-pass filtering is performed in the Radon domain to reduce the high-frequency noise amplified by the Ram-Lak kernel. It is usually implemented by applying a Butterworth or a Shepp-Logan window in the frequency domain. The optimal parameters of the low-pass filter differ from one anatomical region to another. Their preset values are kept fixed in the clinical CT scanners, and the radiologist selects an appropriate one for each clinical study. As a study in [15] shows, the image properties depend nonnegligibly on this parameter.

## 3. Learned Shrinkage in a Transform Domain

 We describe the learned shrinkage algorithm proposed by Hel-Or and Shaked in [12] for signal denoising. A popular Bayesian approach for recovery of a signal *x* from measurements *y* = *x* + *ξ*, contaminated with i.i.d. Gaussian noise, consists in solving the penalized least squares optimization problem,

(6) $$\begin{array}{c} \hat{\alpha }=\arg\underset{\alpha }{\min}{||y-D\alpha ||}_{2}^{2}+\lambda \rho \left(\alpha \right), \end{array}$$

and computing the signal estimate by $\hat{x}=D\hat{\alpha }$ [16]. Effectively, the sought signal is encoded in terms of the dictionary **D** (a linear transform, e.g., wavelets), whose properties encourage noise reduction. The left summand in this expression is a data fidelity term, and the right one expresses expected properties of the signal's coefficients. The classical assumption of a rapid decay of their magnitudes corresponds to the following penalty expression [17, Section 1.3]:

(7) $$\begin{array}{c} \rho \left(\alpha \right)={||\alpha ||}_{p}^{p},\;\text{with}\;\;0\leq p\leq 1. \end{array}$$

For a unitary transform **D**, the problem (6) is separable and admits a simple closed-form solution, which is described by a scalar *shrinkage* function *𝒮* applied elementwise to the vector of coefficients **D** ^−1^ *y*. The formula for the shrinkage function is derived analytically from the expression for *ρ*(*α*) [18]. Thus, the signal estimate $\hat{x}$ has the formula:

(8) $$\begin{array}{c} \hat{x}=D𝒮D^{-1}y. \end{array}$$

This technique was pioneered by Donoho and Johnston [19], who developed it for the wavelet transform, and it is now widely applied in the broader context of nonunitary operators and even redundant dictionaries which are tight frames (see [12, 16] for an overview). In those cases, any shrinkage operation can provide only an approximate solution to (6).

For image denoising, the data dimensions are too large to process the entire image if **D** is not a computationally efficient structured transformation and also, while images vary wildly, small image patches fall into a well-structured statistical pattern. Therefore, the shrinkage idea is applied to an image denoising by extracting overlapping square patches and processing each of them separately. The overlaps help avoiding block artifacts and stabilize the filtering action. Each pixel is altered differently in each patch it belongs to; strong differences are tamed by averaging over all those patches.

Technically, a patch *p* of size *d* × *d*, corresponding to a location *k* in the signal matrix *y*, is extracted by the linear operator **E**_*k*_ and is reinstalled (after a processing) into a signal-sized empty matrix by its transpose **E**_*k*_^*⊤*^. Thus, the patchwise denoising action for a 2D signal is described by

(9) $$\begin{array}{c} \hat{x}=G_{D}\left(y\right)=M_{E}^{-1}\sum_{k}E_{k}^{⊤}D𝒮D^{-1}E_{k}y. \end{array}$$

Here **M**_**E**_ = ∑_*k*_ **E**_*k*_^*⊤*^ **E**_*k*_ is compensating for the overlapping by dividing each pixel by the number of patches containing it.

When the dictionary **D** is a nonunitary full-rank matrix, the vector of coefficients is computed using the pseudoinverse **D** ^+^ of the dictionary. As mentioned before, in this case no exact solution for the shape of the shrinkage function is available. A practical solution for denoising in this setting is proposed in [12]: the shape of the shrinkage function in (9) is learned in an example-based process (rather than being defined descriptively), by optimizing an objective function. Also, for better results, it is preferable to use an array of shrinkage functions, corresponding to the structure of the transform **D**, rather than a single one. For instance, when an *N*-levels wavelet transform is used, separate functions are dedicated to each level. The vector *α* in this case is partitioned into *N* subsets, each processed with an individual shrinkage function.

In [12], the shrinkage functions are modeled as linear combinations of splines of order 1. In other words, these are piecewise linear functions whose joints are configurable. The *N* shrinkage functions *𝒮*_1_,…, *𝒮*_*N*_ are defined by two sets of vectors, **q** = [**q**_1_,…, **q**_*N*_] and **p** = [**p**_1_,…, **p**_*N*_]. The vector **q**_*i*_ is an evenly spaced sequence of numbers, covering the dynamic range of the *i*th subset in *α*; each *𝒮*_*i*_ is the antisymmetric piecewise linear function determined by the following equations:

(10) $$\begin{array}{c} {𝒮}_{i}\left(q_{i}\left(j\right)\right)=p_{i}\left(j\right),\;{𝒮}_{i}\left(-q_{i}\left(j\right)\right)=-p_{i}\left(j\right),\;\forall j\\ {𝒮}_{i}\left(0\right)=0. \end{array}$$

The antisymmetry assumption comes from the belief that only the absolute values of the coefficients should affect the amount of shrinkage applied. It was verified experimentally both in the work of Hel-Or and Shaked and in ours.

The shrinkage operator now has a parameter set **p**, assuming a fixed set of domains **q**. We define an estimator **G**_**p**,**D**_ for the signal *x*, based on (9) with this addition:

(11) $$\begin{array}{c} \hat{x}=G_{p,D}\left(y\right)=M_{E}^{-1}\sum_{k}E_{k}^{Τ}D{𝒮}_{p}D^{+}E_{k}y. \end{array}$$

Let us denote by *α*_*k*_ = (**D** ^+^ **E**_*k*_ *y*) the representation of the *k*th patch in the noisy signal.

The objective function for tuning the parameter set **p** is the mean square error (MSE) of the signal estimate $\tilde{x}=G_{p,D}(y)$, with respect to the true signal *x* available at the training stage:

(12) p∗=argmin⁡p||Gp,D(y)−x||22=argmin⁡p||ME−1∑kEk⊤D𝒮p(αk)−x||22,           αk=D+Eky.

Hel-Or and Shaked define the *slice transform* (SLT) which is applied to *α*_*k*_ in order to reformulate the shrinkage operation as a linear function in **p**. Explicitly, a large sparse matrix *U*_**q**,*α*_*k*__ encoding this data is designed ([12, Section IV]) to perform the shrinkage via a matrix-vector product:

(13) $$\begin{array}{c} U_{q,\alpha_{k}}\cdot q=\alpha_{k},\;\;U_{q,\alpha_{k}}\cdot p={𝒮}_{p}\left(\alpha_{k}\right). \end{array}$$

Using this approach, the optimization of the objective function (12) turns into a simple least squares problem,

(14) $$\begin{array}{c} p^{*}=\arg\underset{p}{\min}{||M_{E}^{-1}\sum_{k}E_{k}^{⊤}DU_{q,\alpha_{k}}\cdot p-x||}_{2}^{2}. \end{array}$$

It is easily solved for **p** using the pseudo-inverse operator.

An application of this method to image denoising, demonstrated in [12], shows very promising results. Moreover, the use of custom-built functions makes the shrinkage operation more robust and suitable for signal processing problems other than noise reduction. For instance, an algorithm for single image super resolution, proposed in [20] and based on the same principles, exhibits a state-of-the-art performance.

## 4. Error Measure in the Image Domain

### 4.1. Constructing the Error Measure

 Before considering an example-based training for CT reconstruction, one must establish a viable error measure in the image domain, minimization of which would lead to radiological images of a good quality. We consider a quantitative error measure for the deteriorated CT image $\tilde{f}$ (reconstructed with some algorithm), which uses the ground truth image *f*. The basic choice for such measure is the mean square error (MSE):

(15) $$\begin{array}{c} \text{MSE}\left(\tilde{f}\right)={||f-\tilde{f}||}_{2}^{2}. \end{array}$$

Pursuing a low MSE value is an accepted goal in the general field of image processing; however, we observe in our experiments that algorithms optimized for minimal MSE produce images with reduced spatial resolution. The reason is that the true image often contains large nearly constant regions, which calls for extensive smoothing. Fine details, washed off by this smoothing, do not increase the MSE notably, so its net value over the entire image is low. We therefore introduce an additional component to the error measure, which encourages the preservation of fine edges in the image.

Consider an edge between two homogeneous regions in the image, where the change in intensity is small comparing to the global dynamic range. If the filter applied blurs this edge, the MSE value is increased by a small amount; however, the gradient norm at the edge is much smaller than in the original image. Thus, it makes sense to penalize not only for difference in intensity values between the reference image *f*_0_ and the reconstruction $\tilde{f}$ but for the difference in gradient norms:

(16) $$\begin{array}{c} Q_{1}\left(f_{0},\tilde{f}\right)=\sum_{x}\left|{||\nabla f_{0}(x)||}_{2}^{2}-{||\nabla \tilde{f}(x)||}_{2}^{2}\right|. \end{array}$$

However, the stated error measure is still reduced by over-smoothing the image: wherever the value of ${||\nabla \tilde{f}(x)||}_{2}^{2}$ is larger than ||∇*f*_0_(*x*)||_2_^2^ (which happens a lot in the noisy image $\tilde{f}$), smoothing the image $\tilde{f}$ will reduce the error. Therefore, we restrict our error measure only to the regions where the original gradient norm is larger than the reconstructed one; those regions are most problematic in the sense of lost details. Thus, the following formula for a penalty component is proposed:

(17) $$\begin{array}{c} Q\left(f_{0},\tilde{f}\right)=\sum_{x}W\left(x\right)\max\left(0,{||\nabla f_{0}\left(x\right)||}_{2}^{2}-{||\nabla \tilde{f}\left(x\right)||}_{2}^{2}\right). \end{array}$$

The weight matrix *W*, introduced here, is designed to remove all the locations where the reference gradient norm is above 2% of its maximal value. This (empirically chosen) threshold is applied in order to focus on the low-contrast edges and not to waste the learning capacity of the filter on the strong flesh-bone transitions. For a practical optimization we replace the function (max⁡(0, *x*) has a noncontinuous derivative in 0) max⁡(0, *x*) with a smoothed version *ψ*(*x*, *δ*), based on the Huber penalty function [21]:

(18) $$\begin{array}{c} \psi_{\delta }\left(x\right)=\{\begin{array}{cc} 0, & x<0\\ \frac{x^{2}}{2}, & 0\leq x<\delta \\ \delta |x|-\frac{\delta^{2}}{2}. & x\geq \delta . \end{array} \end{array}$$

Another modification to the formula is introduced. Often the radiologist is primarily interested in observing the clinical images in a specific dynamic range; for instance, if soft tissues are of interest, the relevant range seldom exceeds the window of [−300,300] Hounsfield units (HU). On other hand, if bones are observed, the range should cover the bone intensity; in this case, a range of [0,1500] HU may be relevant. We provide the ability to use this information in order to concentrate the learning capacity of the filter in the required dynamic range. Thus we introduce a binary mask **H** in the image domain, which (in the training stage) is set to exhibit only those image regions which fall into the relevant dynamic range. Bottom line, we set the main penalty component to the form of weighted *L*_2_ norm, ${||f-\tilde{f}||}_{2,H}^{2}={\left(f-\tilde{f}\right)}^{T}H(f-\tilde{f})$. The obtained error measure is named MSEg (mean square error, augmented with the g gradient), and its final expression is

(19) $$\begin{array}{c} \text{MSEg}\left(f_{0},\tilde{f}\right)={||f_{0}-\tilde{f}||}_{2,H}^{2}+\mu Q\left(f_{0},\tilde{f}\right),\\ \text{where}\;\;Q\left(f_{0},\tilde{f}\right)=\sum_{x}W\left(x\right)\psi_{\delta }\left({||\nabla f_{0}(x)||}_{2}^{2}-{||\nabla \tilde{f}(x)||}_{2}^{2}\right). \end{array}$$

The reference image *f*_0_ will be omitted from the notation from now on. Using this function for training of data filters produces CT images, which combine low MSE value with high spatial resolution measure. In the following principle experiment, it is compared to the basic MSE penalty in order to demonstrate the effect of the proposed gradient-based term.

### 4.2. Empirical Evaluation of the Proposed Measure

**Figure 1:**
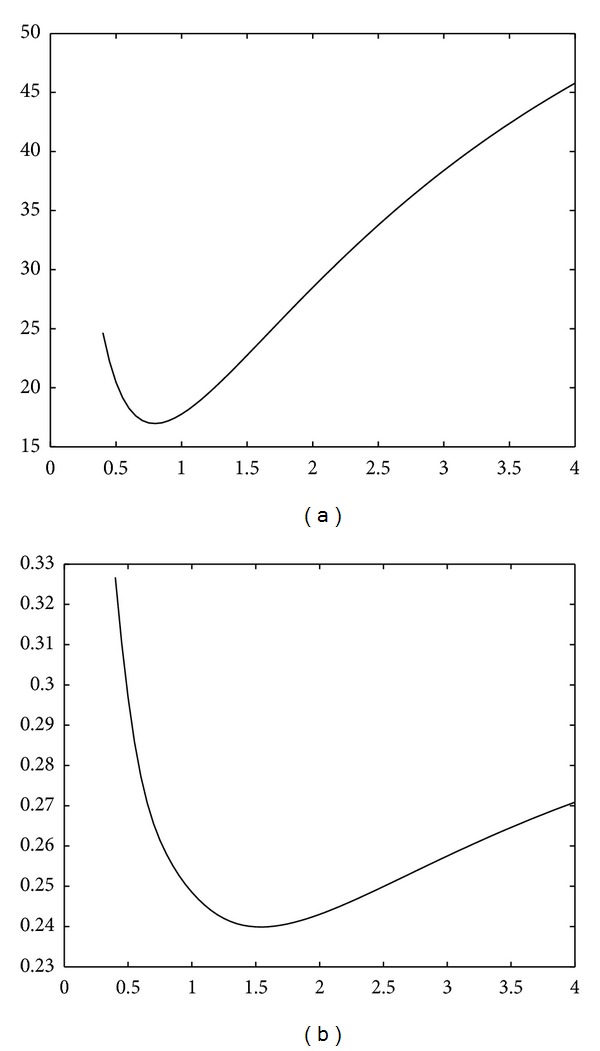
Graphs of the MSE values ${||f_{0}-\tilde{f}||}_{2}^{2}$ (a) and the gradient penalty values $Q(f_{0},\tilde{f})$ (b) as a function of the cutoff frequency.

**Figure 2:**
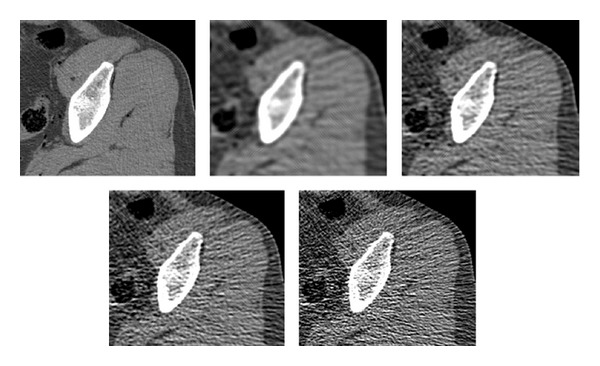
Left to right, upper to lower: reference image, FBP reconstructions obtained with low-pass filter with *ϕ*_0_ = 0.4 (very low), *ϕ*_0_ = 0.8 (optimal for MSE), *ϕ*_0_ = 1.55 (optimal for gradient-based measure $Q(f_{0},\tilde{f})$), and *ϕ*_0_ = 4 (very high).

 The following experiment shows the effect of the gradient-based term in (19) on the visual impression from the “optimized” image. The FBP algorithm is employed to perform CT reconstruction from noisy observations. The cutoff frequency *ϕ*_0_ of the low-pass filter in the Radon domain is gradually increased to alter the variance-resolution tradeoff in the reconstructed image. In Figure 1 we display a graph of MSE values of the obtained image as a function of *ϕ*_0_ and a graph of $Q(f_{0},\tilde{f})$ values. Both are unimodal graphs with a single minimum. For $Q(f_{0},\tilde{f})$, the minimum is obtained at a higher frequency than for the MSE measure. In Figure 2 we display reconstructions corresponding to these two optimal frequencies. A visual inspection led us to the conclusion that the minimal-MSE version (upper row, on the right) is a blurred image, where spatial resolution is sacrificed for noise reduction. The version minimizing the $Q(f_{0},\tilde{f})$ penalty (lower row, on the left) is visually more appealing since it has a higher spatial resolution at the expense of stronger noise that is managed well by the human eye. For comparison, we also display the reference image (upper left) and two extreme cases corresponding to low/high cutoff frequency (middle column). 

 The two conclusions drawn from this experiment are (1) if we add the gradient-based penalty $Q(f_{0},\tilde{f})$ to the error measure and train the reconstruction chain to minimize its value, the obtained images are more informative to the human eye, with higher spatial resolution, and the cost is a higher noise level and (2) the values of $Q(f_{0},\tilde{f})$ are lower by two orders of magnitude than the MSE; therefore, in a balanced total error term (19), the value of the weight *μ* should be around 100 in order for $Q(f_{0},\tilde{f})$ to have an effect.

## 5. The Algorithm for CT Reconstruction

The algorithm consists of the sequence of steps, producing a CT image from the measured photon counts. They are stated here and detailed in the sequel.

- *Data adjustment for signal processing*: the photon counts are altered so as to enable an approximate modeling with only the Poisson random variable; then, they undergo the Anscombe transform [22] to normalize the noise variance.
- *Learned Shrinkage in the measurements domain:* the 2D matrix of the adjusted measurements data is processed patchwise using the learned shrinkage algorithm (we modify the original method of Hel-Or and Shaked, adjusting it to indirect measurements).
- *Standard FBP:* the FBP transform with no low-pass filter reconstructs the CT image from the restored measurements data.
- *Learned Shrinkage in the image domain:* a different instance of the learned shrinkage algorithm is applied on the obtained image, producing the final outcome.

We now extend the discussion on each of these steps.

### 5.1. Adjusted Measurements

 The first step is to remove the Gaussian component from the measurements model stated in (1). Following [1], we compute the adjusted variables $\hat{y}$:

(20) $$\begin{array}{c} {\hat{y}}_{\ell }={\left[y_{\ell }-d+\sigma_{n}^{2}\right]}_{+}, \end{array}$$

where the [*x*]_+_ = max⁡{*x*, 0}. It is easily seen that the expectation and the variance of this distribution are equal to those of the single Poisson variable with the parameter ${\hat{\lambda }}_{\ell }=\lambda_{\ell }+\sigma_{n}^{2}$, and so for the purposes of noise normalization, we will assume that this is the distribution modeling for the adjusted measurements ${\hat{y}}_{\ell }$. Also, we assume a zero-mean electronic noise (*d* = 0), and therefore the positivity correction is not relevant.

The variance of the Poisson random variable equals its expectation ${\hat{\lambda }}_{\ell }=\lambda_{\ell }+\sigma_{n}^{2}$ and can be approximated by the measured value ${\hat{y}}_{\ell }$. We assume that the noise reduction by the learned shrinkage works best when the noise is homogeneous (constant variance in all points). The reason is that the representations of all the patches in the data matrix are processed by the same scalar function, and if the noise energy in each patch was different, it would require a spatially varying shrinkage. In order to achieve unit variance in all the measurements, we use the Anscombe transform [22], intended to normalize the Poisson variable:

(21) $$\begin{array}{c} \phi \left(x\right)=2\sqrt{x+\frac{3}{8}}. \end{array}$$

To summarize, the overall data adjustment is performed elementwise by the following scalar function *ω*(*x*):

(22) $$\begin{array}{c} z_{\ell }=\omega \left(y_{\ell }\right)=\phi \left(y_{\ell }+\sigma_{n}^{2}\right)=2\sqrt{y_{\ell }+\sigma_{n}^{2}+\frac{3}{8}}. \end{array}$$

### 5.2. The Objective Function for Training

 Let *z* = *ω*(*y*) denote the matrix of adjusted measurements. We state the expression for the image $\tilde{f}$ reconstructed with our algorithm, before the postprocessing stage. The noise reduction in *z* is done by the nonlinear filter **G**_**p**_ = **G**_**p**,**D**_, defined in (11). Here the dictionary **D** is a fixed linear transformation (we use the unitary discrete cosine transform), and therefore it is omitted from the notation. After the filtering, the data is transformed to the Radon domain by applying the *ω* ^−1^(*x*), followed by the −log⁡ function. Then the FBP operator **T** is applied to produce the CT image $\tilde{f}$. To summarize, the image is computed as follows:

(23) $$\begin{array}{c} {\tilde{f}}_{p}\left(z\right)=-T\left(\log\left(\frac{1}{\lambda_{0}}\omega^{-1}G_{p}\left(z\right)\right)\right). \end{array}$$

The objective function for the training of the parameter set **p** is the proposed error measure from (19), regularized with an additional factor:

(24) Γp(z)≡MSEg(f,f~p(z))+γ||p−q||2=||f−f~p(z)||2,H2+μQ(f0,f~p(z)) +γ||p−q||2.

Here *γ*||**p**−**q**||_2_ is a regularization term penalizing the deviation of each shrinkage function from the identity. Its purpose is to make the shape of the shrinkage functions more robust to outlier examples.

In order to minimize the function Γ_**p**_(*z*) with respect to the argument **p**, we use the memory-efficient *ℓ*-BFGS convex optimization method [23], which requires computing the value and the gradient of the function being minimized. The implementation of the algorithm is by the courtesy of Mark Schmidt (see http://www.di.ens.fr/mschmidt/Software/minFunc.html). Since our objective function is not convex, there is a theoretical question regarding the convergence of this numerical scheme. In practice, we have observed that the method converges in ~100 iterations, and the obtained parameter set enables producing CT images of a good quality (in comparison to other reconstruction methods).

The gradient of the function **G**_*p*_(*z*) (defined in (11)) with respect to **p** can be expressed with the help of the slice transform proposed in [12]. Recall that *α*_*k*_ = **D** ^+^ **E**_*k*_ *z* is a representation of the *k*th patch in *z*. The shrinkage operation *𝒮*_**p**_ *α*_*k*_ is replaced by an equivalent matrix-vector multiplication *U*_**q**,*α*_*k*__ **p**. Then **G**_*p*_(*z*) has the form

(25) $$\begin{array}{c} G_{p}\left(y\right)=M_{E}^{-1}\sum_{k}E_{k}^{⊤}DU_{q,\alpha_{k}}p, \end{array}$$

which is linear in **p**. Now, if we consider the estimator ${\tilde{f}}_{p}(z)$ as a function of $\tilde{z}=G_{p}(z)$, we see that this is a composition of elementwise scalar function −log⁡(1/*λ*_0_)*ω* ^−1^ and the linear operator **T**. Thus, the gradient here is also easily computed. Finally, the gradient of the functional MSEg with respect to $\tilde{f}$ consists of *L*_2_ norms and derivative operators, so its expression is derived using standard methods. We conclude that the gradient ∇_**p**_Γ_**p**_(*z*) has a closed-form analytical expression and can be readily computed.

The expressions presented above involve only one image *f*, just for a better readability; in practice, we may sum the errors over a training set of many images *f*.

Another important remark is this: when this training objective is used, the parameters of the shrinkage operator *𝒮*_**p**_ are tuned to not to reduce the photon count noise in the measured data *y* (via the processing of the adjusted data *z*) but to prepare the data in the best way for the specific reconstruction operator **T**. Here lies the key difference between this algorithm and existing methods for measurements denoising, which target high signal quality in the raw data but do not consider the final CT image.

### 5.3. Image Postprocessing

 At this point, after the pre-processing filter is tuned, we have a working reconstruction chain that produces CT images. The stated objective is pursued by its components somewhat indirectly, by changing the raw measurements. A further reduction of the reconstruction error can be achieved by administering another filter, acting on the obtained CT images themselves. We use again the method of learned shrinkage. The training data comprises of the set of CT images ${\tilde{f}}_{p}(z)$, reconstructed from the original noisy measurements, as described before. The corresponding reference images serve again as the ground truth. Overlapping patches are extracted from an image, processed by the shrinkage functions in the transform domain and are reinstalled back. The noise statistics in the obtained CT images are difficult to estimate because of the preprocessing stage. Therefore, we do not attempt to normalize the noise in the image patches.

We formulate the training objective for the parameter set **p** ^*I*^ of the image domain shrinkage similarl to the case of measurements domain:

(26) ΓpII(f~)=MSEg(f,GpI(f~))+γI||pI−qI||2=||f−GpI(f~)||2,H2+μQ(f0,GpI(f~)) +γI||pI−qI||2.

Here the upper script *I* denotes the image domain. The input image $\tilde{f}$ is computed via the formula in (23) using the vector **p** of shrinkage parameters, learned earlier. This way the postprocessing is tuned for the very same kind of images it will be getting in the operational mode. As previously stated, the training stage consists of minimizing the value of $\Gamma_{p^{I}}^{I}(\tilde{f})$ with respect to **p** ^*I*^. This task is simpler than the optimization in the measurements' domain since no data adjustment or reconstruction is required. Using an expression for the gradient ∇_**p**^*I*^_Γ^*I*^, we invoke again the *ℓ*-BFGS method to solve the optimization problem. The convergence here is faster than in the measurements domain, and it takes about 30 iterations to the convergence.

We remark that the postprocessing method could be evaluated in its own right, when the corrupted input images may come from the standard FBP or from any other reconstruction method. This evaluation is left for a future study.

### 5.4. The Training Set

 The example-based training approach requires a collection of high quality reference images, each accompanied with a degraded set of measurements. It is preferable to compose the training set from clinical images obtained from a CT scan of a human body, rather than from images of synthetic phantoms. In any case, the example object has to be scanned twice: one time with a very high X-ray dose to compute a high-quality reference image (using standard FBP, for instance), and another time with the low dose desired for the practical scan performed on patients. This configuration is feasible with human cadavers: there is no restriction on the X-ray dosage, and there is no problem of registration between the two consecutive scans for a still object (in our experience, with a clinical scanner of General Electric). Another approach for producing the training pairs is to start with given high-quality CT slices and simulate the low-dose measurements by reproducing the machine's X-ray operation as faithfully as possible. This is the approach we took in our work.

We suggest that the training set should be composed of CT images representing a specific anatomical region. This is in light of the observation that the characteristics of CT images vary substantially between different such regions. This leads to building a collection of learned parameter sets, specialized for head, lungs, abdomen, arms, and so forth. During the reconstruction, the operator of the CT scanner should choose the relevant version of the parameter set, just like it is done today with the different smoothing filters for the standard FBP. Nevertheless, in our numerical experiments we also show that a filter specialized on one anatomic region also makes a good performance on other ones. A deeper study of dependence of the learned filter on the training set should be carried out by professional radiologists.

A training set we build for a specific anatomic region consists of a sequence of axial slices from that region, uniformly distributed in *z*-axis. The size of the training set is also a parameter to be investigated; we found that 9–12 images suffice for a stable optimization and a consecutive robust reconstruction of the thighs or the abdomen regions. However, in regions rich with small details where there are important but subtle differences between nearby slices (the brain, for instance) a larger collection of images is possibly required.

### 5.5. Computational Complexity

 The number of operations required for the *n* × *n* image reconstruction with our algorithm is *𝒪*(*n* ^3^), which is the same complexity as required by the regular FBP alone.

The measurements' matrix consists of $2n\times \sqrt{2}n=2.82n^{2}$ elements. The learned shrinkage applied to the measurements consists in applying the analysis operator **D** ^+^ at each patch of size *d* × *d*, then applying the scalar shrinkage function on each of the *d* ^2^ coefficients and recomputing the patch by a multiplication with **D**. The dictionary **D** we use is the unitary 2D discrete cosine transform (DCT), which requires *𝒪*(2*d* ^2^log⁡(*d*)) operations. We use patches of size *d* = 11; thus, computing a representation of each patch requires about 2*d* ^2^log⁡(*d*) = 580 operations and the same amount of work to convert the representation back to the signal. Further, applying a shrinkage function takes *𝒪*(*c* · *d* ^2^) operations, where the constant *c* is about 20, governed by the length of the vector **q** in (10). The number of patches, extracted from an *n* × *n* image, depends on the chosen amount of overlapping; up to *n* ^2^ patches can be processed. To summarize, the application of the learned shrinkage filter has the computational complexity of *𝒪*(4*c* · *n* ^2^ *d* ^2^(log⁡(*d*) + 1)). The standard size of clinical CT images is *n* = 512, so the number *d* ^2^(log⁡(*d*) + 1) is of the same order of magnitude as *n* (for *d* = 11, this number equals 822). Therefore, it takes about 80*n* ^3^ operations, which is comparable to the *𝒪*(*n* ^3^) complexity of the FBP transform.

We notice that the processing of individual patches can be naturally parallelized on multiple core or GPU, reducing the computation time by a nonnegligible factor (depending on the available hardware).

## 6. Empirical Study

### 6.1. Experimental Setup

**Figure 3:**
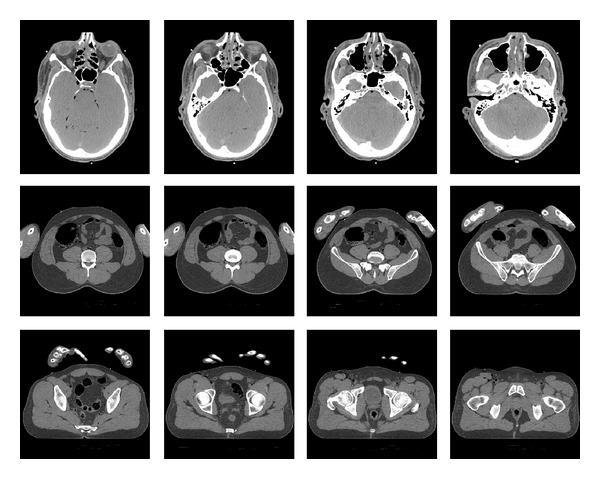
Examples of clinical images used in the experiments. Upper row: axial head slices displayed in the range (HU window) of [−170,250]), middle row: abdomen images, lower row: thigh images. The two lower rows are displayed in the HU window [−220,350]. Head images are slightly enlarged relative to other regions for better visibility.

 The algorithm was implemented using the Matlab environment and tested on sets of clinical CT images (axial slices). These were extracted from a CT scan of a male, from regions of the head, abdomen, and thighs. The images are courtesy of the Visible Human Project (See http://www.nlm.nih.gov/research/visible/visible_human.html). The images are of dimensions 512 × 512, acquired with 1 mm intervals along the *z*-axis. The intensity levels correspond to Hounsfield units (HU), given with the accuracy of 12 bits per pixel. Representatives of these sets we have assembled are displayed in Figure 3. We wish to point out that the reference images used for the training stage are not perfect since they were obtained using a standard X-ray dosage. If very-high-quality images were available for the training, we would expect our algorithm to perform better.

In absence of raw measurements' data from a CT scanner we simulate the scan process by computing projections of given CT images (considered to be the ground truth) as follows. First, the intensity values in the image are converted from the Hounsfield units to the units of reciprocal length, corresponding to the linear attenuation coefficient *μ*. The relation between the two scales is (http://www.medcyclopaedia.com/library/topics/volume_i/h/hounsfield_unit.aspx/)

(27) $$\begin{array}{c} \text{HU}\left(x\right)=\frac{\mu \left(x\right)-\mu \left(\text{water}\right)}{\mu \left(\text{water}\right)-\mu \left(\text{air}\right)}\cdot 1000, \end{array}$$

where *μ*(water) = 0.19 cm^−1^, *μ*(air) = 0 cm^−1^. The original 512 × 512 images are cropped to dimensions 461 × 461 by removing the empty background (to save computation time). Then, noiseless sinogram $\overset{-}{g}=Rf$ is simulated by applying to the reference image a pixel-driven implementation of the discrete 2D Radon transform. The algorithm for forward- and back projection uses linear interpolation in the locations of bins/pixels. Explicitly, each bin in a projection is a weighted sum of a few (temporary) finer bins, which are computed by integrating image intensities over a narrow (quarter of a pixel) ray in the image domain. The weights are linear in the distance between the center of the coarse bin and the centers of the fine bins.

For *n* × *n* images, we have used *n* views (projections), evenly distributed over the angle range [0, *π*]. Each projection consists of $\sqrt{2}n$ bins. Ideal photon counts are computed from the sinogram entries via the relation $\lambda_{\ell }=\lambda_{0}e^{-{\overset{-}{g}}_{\ell }}$. The measured photon counts *y*_*ℓ*_ are produced by generating random Poisson variables with expectations *λ*_*ℓ*_ and zero-mean Gaussian variables with a chosen standard deviation *σ*_*n*_. The X-ray dose is controlled by the maximal photon count *λ*_0_ and the value of *σ*_*n*_.

The design parameters of the proposed algorithm are set as follows. The filter, based on the learned shrinkage, is implemented using the 2D unitary discrete cosine transform (DCT) (see Figure 5). It is implemented using *d* ^2^ elements composing a linear basis, which are matrices of dimensions *d* × *d*. The representation of a *d* × *d* signal is computed as the set of inner products between the signal and each basis element. This approach allows computing the representation of the entire collection of image patches in a batch, by convolving the image with each basis element. In our work we set *d* = 11.

**Figure 4:**
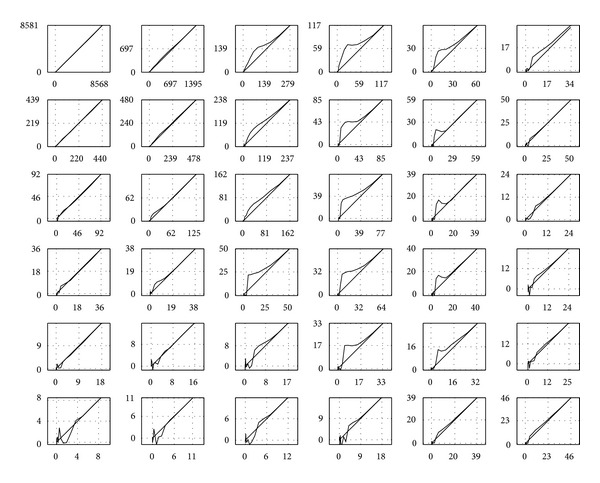
Shrinkage functions obtained via the learning process on a training set consisting of male thighs images. Only the odd rows and columns from the original 11 × 11 array of functions are displayed due to space considerations. Due to the antisymmetry, only the positive half of the *x*-axis is drawn.

**Figure 5:**
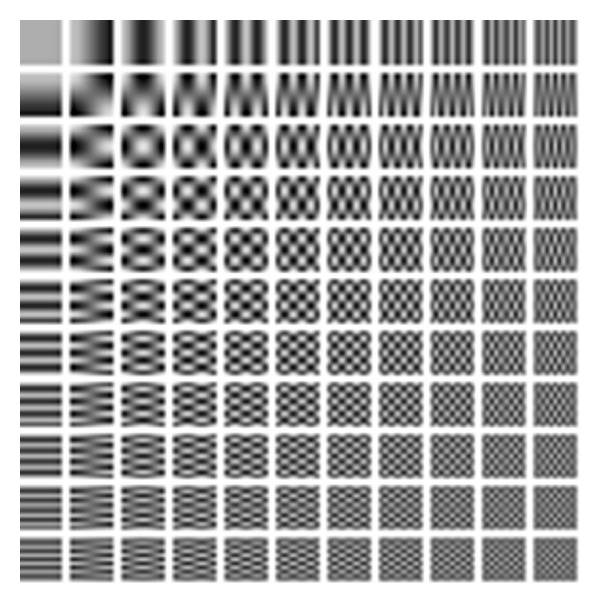
Two-dimensional discrete cosines basis. Each square in the 11 × 11 array is a 2D function representing a basis element.

Each of the 121 corresponding shrinkage functions of the operator *𝒮*_**p**_ consists of 2 × 20 linear pieces (the factor of 2 is for the negative and the positive parts; recall that the shrinkage functions are antisymmetric by design, so there is just 20 degrees of freedom); this number was established empirically and is similar to the one used in [12]. Graphs of shrinkage functions, obtained in one of the training sessions, are displayed in Figure 4. The regularization parameters in (24), (26) were set to *γ* = 10^−4^, *γ*_*I*_ = 250. Use of the regularization has helped to constrain the shapes of the pre- and postprocessing shrinkage functions, avoiding jumps resulting from outlier samples. We discuss the process of their tuning in the sequel.

We mention that numerical experiments were also carried out with the nondecimated 3-level Haar Wavelet frame, but they are not presented here due to slightly inferior results (comparing to those obtained with the DCT). However, our impression is that a particular choice of the transform is not of a crucial importance. Another remark we shall make concerns the redundancy of the dictionary. We have compared the performance of the algorithm using the unitary DCT against its version with an overcomplete DCT. In our experiments, no improvement was induced by this change.

### 6.2. Implementation of the Existing Algorithms

 We implement three existing reconstruction algorithms. First is the standard FBP, with the classical noise reduction done by a sinogram smoothing. Second is an iterative, statistically-based algorithm, approximating the penalized likelihood solution; specifically, it is the penalized weighted least squares (PWLS), proposed in [6]. The third method is the adaptive truncated median (ATM) filter [8]. It is close in its spirit to our work—a fast nonlinear preprocessing method, designed to improve the performance of FBP in a low-dose scan scenario.

#### 6.2.1. Implementation of the FBP Algorithm

 The FBP was realized according to the following formula:

(28) $$\begin{array}{c} T=R^{*}\circ F_{\text{low}}\circ F_{\text{RL}}. \end{array}$$

**F**_RL_ is a convolution filter with the discrete Ram-Lak kernel *κ* of *m* taps, computed via the following formula ([24, equation (5.5)]):

(29) κ(p)=sinc(p)−12(sinc(p2))2, p=−m2:m2,sinc(x)=sin⁡(x)x.

The low-pass filter **F**_low_ is implemented by composing the Ram-Lak filter with the Butterworth window [25] in the Fourier domain. An expression for this window is

(30) $$\begin{array}{c} |H\left(\omega \right)|={\left(1+{\left(\frac{\omega }{\phi_{0}}\right)}^{2p}\right)}^{-1/2}. \end{array}$$

The two defining parameters are the cutoff frequency *ϕ*_0_, which controls the frequency response of the filter and the order *p*, which affects the steepness of its roll-off. In the experiments, these two parameters are tuned manually for the best visual impression on the training set (this issue is discussed in the sequel).

#### 6.2.2. Implementation of the PWLS Algorithm

 We used the objective function stated in Equation (14) of [6], which represents an approximation to the log-likelihood function of the CT image $\tilde{f}$, with an addition of a penalty component $R_{\delta }(\tilde{f})$. In our notation it is stated as follows:

(31) L(y ∣ f~)=12∑ℓWℓ([Rf~]ℓ−gℓ)2+γRδ(f~),  whereWℓ=yℓ2yℓ+σn2, gℓ=−log⁡yℓλ0. 

The expression for the regularization is

(32) $$\begin{array}{c} R\left(\tilde{f}\right)=\sum_{p}\;\sum_{k\in 𝒩(p)}\psi \left({\tilde{f}}_{p}-{\tilde{f}}_{k}\right). \end{array}$$

Here *ψ*(*x*) is the convex edge-preserving Huber penalty

(33) $$\begin{array}{c} \psi \left(x,\delta \right)=\{\begin{array}{cc} \frac{x^{2}}{2}, & x<\delta \\ \delta \left|x\right|-\frac{\delta^{2}}{2}, & x\geq \delta \end{array} \end{array}$$

and *𝒩*(*p*) is the set of the four nearest neighbors of *p*. Parameters *γ*, *δ* of the penalty component were tuned manually for best the visual impression on training images.

#### 6.2.3. Implementation of the ATM Filter

 The ATM filter was briefly described in the introduction. Technically, its action on a location *p* in a signal *S* is defined by two parameters: the number *M* of data values in the neighborhood of *p*, involved in the filtering and the fraction *α* of outliers assumed in this data. The filtered value at *p* is computed as the average of this neighborhood, taken after removing the *Mα* highest and the *Mα* lowest values. This filter is made adaptive by using data-dependent parameters *M* and *α*, computed for each location of the measurements matrix. The formulas for ATM parameters, given in [8], involve the signal value (photon count) *x* = *S*(*p*):

(34) $$\begin{array}{c} M=\frac{2\beta \lambda }{2\lambda +\max\left(0,x-\delta \right)},\;\;\alpha =\frac{\alpha_{m}x}{\lambda }. \end{array}$$

In [8] it is not specified what is the shape of the neighborhood of the pixel *p*, involved in its filtering. In our implementation, we assume it is a discrete disc. Also, no method for computing the inner parameters *β*, *λ*, *δ*, *α*_*m*_ is proposed; a set of prescribed values is given instead. In our implementation, these parameters are tuned to minimize the net MSE on the training set, by sweeping two-dimensional grids, built for different pairs of parameters. This is done in iterations, each time a different pair out of the four parameters is changed.

### 6.3. Visual Evaluation and Comparison of the Algorithms

**Figure 6:**
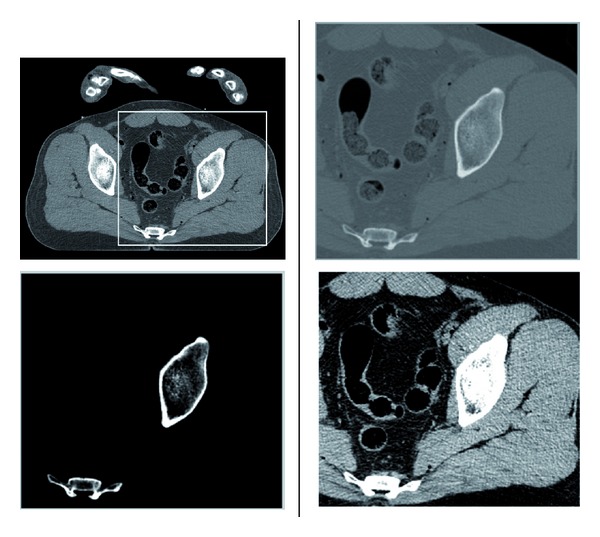
Left to right, upper to lower: test image with a marked zoom-in window, a zoom-in region depicted in dynamic ranges [−1000,1500], [100, 700], [−100,150] HU, respectively. Yet another version of this image appears in Figure 7 (upper left), in [−220,350] HU.

**Figure 7:**
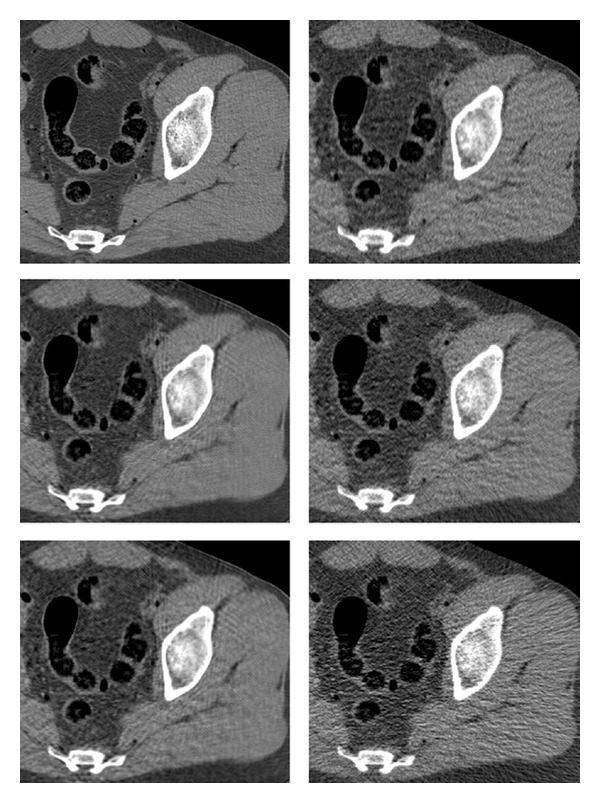
Reconstruction of a test image (thighs' section). Images are listed left to right and displayed in HU range of [−220,350]. Upper row: reference image, PWLS. Middle row: our method (stage II), ATM. Lower row: our method (stage I), FBP.

 The existing and proposed methods are compared on the reconstruction of a test image of thighs' section. The noise on the projection data was generated by setting *λ*_0_ = 1.5 · 10^5^, *σ*_*n*_ = 5. The visual comparison of is given in Figure 7, where the corresponding reconstructed images are presented. We display an enlarged region of the image, for better observability. The displayed dynamic range is set to [−220,350] Hounsfield units (HU), chosen for best visualization of the particular axial slice. In general, there is no predefined HU-window radiologists use to look at the CT images; the window is tuned manually in an interactive fashion, and it depends on the anatomical region, diagnostic purposes, and personal preferences. The reference image is depicted in Figure 6 in few different windows to display the effect of such tuning.

The FBP image (Figure 7, lower right) suffers from streak artifacts, which corrupt its content—especially, the fine details. The strength of the artifacts can be reduced at the cost of blurring the image; here the strength of the low-pass filter in FBP was tuned manually for visually plausible images. The ATM algorithm (middle right) displays a significant reduction in the strength of the streaks, which implies that most outliers in the Radon domain were removed. However, some residual noise and detailed corruption are still present.

The slow, iterative PWLS algorithm (upper row, right) provides a better version of the image, with improved sharpness and complete lack of streaks. The latter property is expected since the image is built as a MAP optimizer and there is no smear of the raw-data errors by the back projection. Still, there is a noise texture in the image. The PWLS performance can be tuned by varying the penalty weight, Huber parameter, and the number of iterations. These parameters were set manually to produce a high spatial resolution, at the cost of manageable noise; specifically, we used 90 iterations, and the value *γ* = 8 · 10^−5^ in (31). Different, MSE-optimized reconstructions by the compared methods are given in Figure 16.

Finally, we refer to the two images produced by our method. The stage-I image (lower left) was obtained by applying the FBP to the preprocessed data. The postprocessing of this image results in the stage-II version (middle left). The FBP streaks are significantly reduced in stage-I image, similarl to the ATM method; however, some of the streaks are sharply visible around the bone area. The reason for their appearance is that the filter is not removing all of the problematic streak effects; since our method is based on FBP, some of its artifacts remain. Moreover, they are better visible on resulting the image due to reduced background noise and lack of the rest of the streaks. We should note that the general noise level is visibly lower than that appearing in the rest of the images. The noise is further reduced in stage-II image, without introducing additional blur.

**Figure 8:**
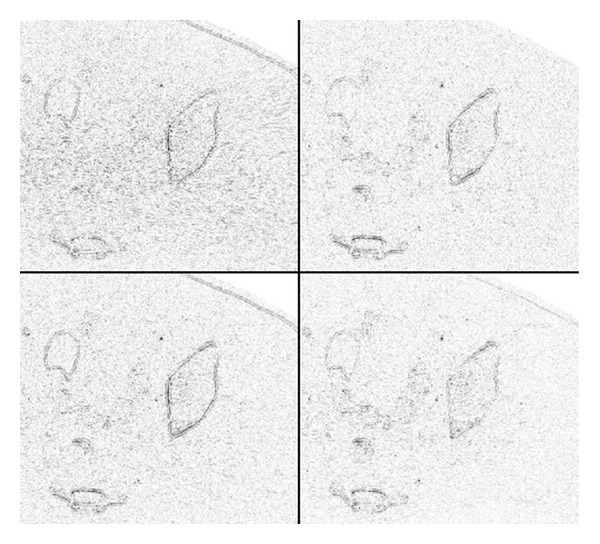
Absolute-valued differences between the reconstructions and the reference image. Darker shade corresponds to a larger error. Left to right, upper to lower: FBP, PWLS, ATM, and our method (stage II).

The error images are displayed in Figure 8. With our method (stage II), the error image has less of the uniform noise than any of the three compared methods, and the edges appearing in the error image (they point to the loss of spatial resolution) are as weak as those observed in the PWLS reconstruction.

In Table 1, some quantitative measures of reconstruction quality are provided. The signal-to-noise Ratio (SNR) is defined for the ideal signal *f* and a deteriorated version $\hat{f}$ by SNR$\left(f,\hat{f})=-20\;\text{lo}{\text{g}}_{10}({||f-\hat{f}||}_{2}/{||f||}_{2}\right)$. In practice, we consider the signal $\hat{f}$ up to a multiplicative constant and compute

(35) $$\begin{array}{c} \text{SNR}\left(f,\hat{f}\right)=\underset{\alpha }{\min}-20\;\log_{10}\left(\frac{{||f-\alpha \hat{f}||}_{2}}{{||f||}_{2}}\right). \end{array}$$

The structured similarity (SSIM) measure was introduced in [26] as an alternative to MSE which is more relevant to human perception of the images. The explicit formula involves first and second moments of the local image statistics and the correlation between the two compared images. In our numerical experiments, we have used the Matlab code provided by the authors of [26], which is available at http://www.cns.nyu.edu/~lcv/ssim/.

Finally, the MSEg measure is introduced in this paper, (19).

The SNR and MSEg values are measured in the range of [−220,350] (HU). The MSEg value is detailed as a sum of the MSE component and the gradient-based component in order to show the balance between the two factors in the different cases. All three of the computed measure consistently point to the gradual quality improvement as one passes from FBP to ATM and to PWLS and finally to the proposed method. 

**Figure 9:**
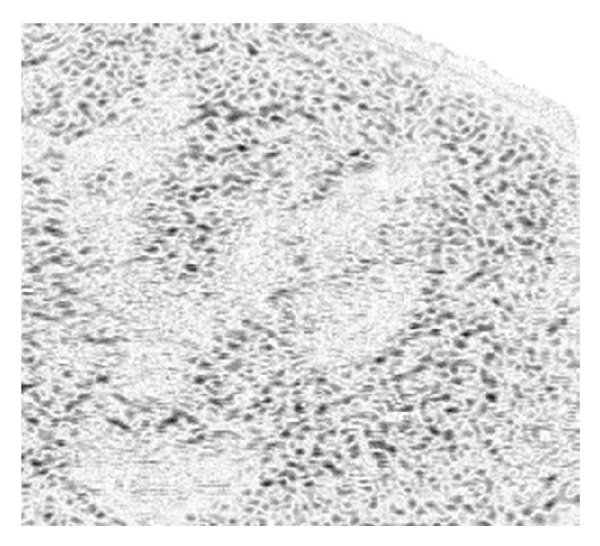
Absolute-valued difference between stage-I and stage-II of our method.

To appreciate the effect of the postprocessing (stage II of our method), we display the absolute-valued difference between the two stages in Figure 9. Almost no structure can be observed in this error image, and this implies that very little of the image content is lost during the postprocessing. 

### 6.4. Behavior in Different Anatomical Regions

**Figure 10:**
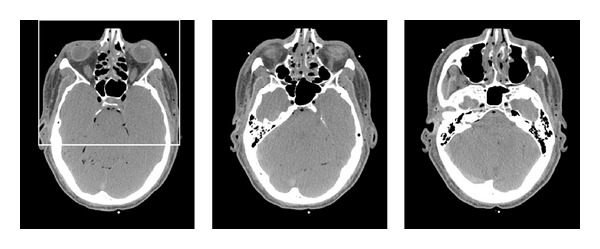
Few example images of head sections. The marked region in the test image (on the left) is zoomed on in Figure 11.

**Figure 11:**
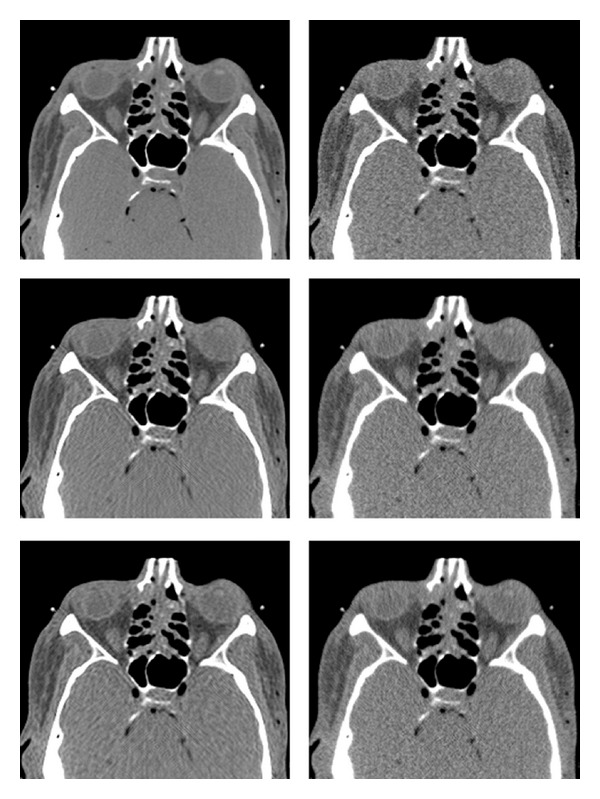
Head reconstruction. Images are listed left to right and displayed in HU range of [−170,250]. First row: reference image, PWLS. Second row: our method (stage II), ATM. Third row: our method (stage I), FBP.

**Figure 12:**
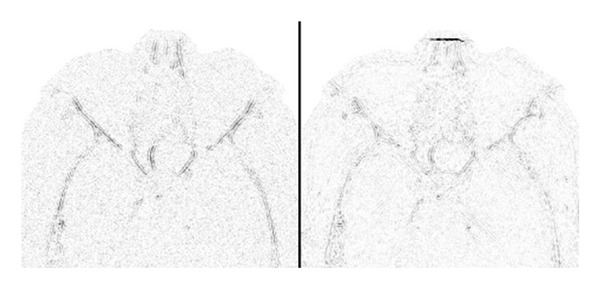
Head reconstruction errors with respect to the reference image (darker shade corresponds to a larger error). Left: PWLS, right: our method (stage II).

**Figure 13:**
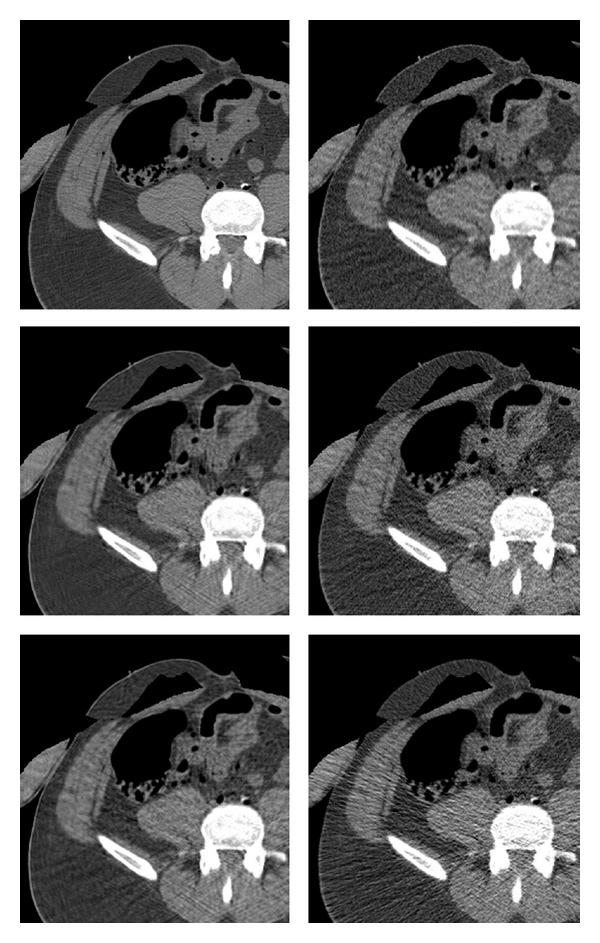
Abdomen reconstruction. Images are listed left to right and displayed in HU range of [−220,350]. First row: reference image, PWLS. Second row: our method (stage II), ATM. Third row: our method (stage I), FBP.

 An important issue of the example-based training approach is the dependence of the reconstruction quality on the training set and the anatomical region. Recall that the reference images for training were taken from the thighs' region; we now use the trained shrinkage functions to restore axial head and abdomen sections. The compared reconstruction methods are also applied (without changing their parameters) to the new regions. The results are displayed in Figures 10–13. The test image from the head region, along with some other examples of head sections, is given in Figure 10. In the FBP reconstruction (Figure 11, lower right), the quality of the fine details is reduced due to the streak noise; both PWLS (upper right) and our method (middle and lower left) exhibit better restoration of these details (see, for instance, the thin vein-like lines on the left and right sides of the image, as well as the small bright spots in the upper central region). With our method the noise texture still exhibits streaks (since this is an FBP-based method), but the noise energy is lower than that in the PWLS, which is reflected in higher SNR value. The parameters of the ATM method should apparently be different for the head scan data since the resulting image is almost the same as the FBP without preprocessing. We conclude that ATM is sensitive to the choice of an anatomical region and has to be tuned using relevant training sets. Quantitative measures, presented in Table 1, also show the similarity between FBP and ATM images and point to improved quality of images produced by the shrinkage method. An exception is the MSEg measurement in the thighs' image, where the PWLS achieves a lower value of the gradient-based penalty.

**Figure 14:**
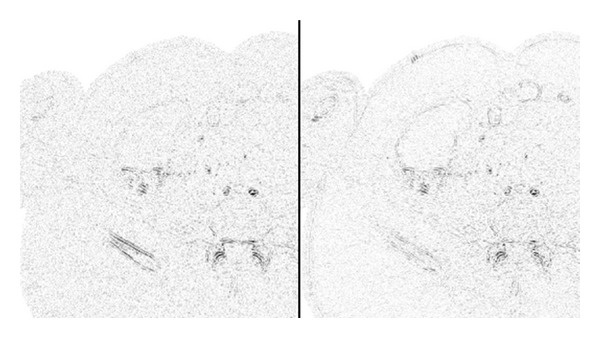
Abdomen reconstruction errors with respect to the reference image (darker shade corresponds to a larger error). Left: PWLS, right: our method (stage II).

In the abdomen image reconstruction (Figure 13) the ATM performs better than the FBP but both ATM and PWLS do not succeed to reduce the noise like the shrinkage method does. Figures 12 and 14 depict the reconstruction error for the PWLS and our algorithm (other methods are omitted here out of space economy) and support the observations above.

### 6.5. Behavior at Different Noise Levels

**Figure 15:**
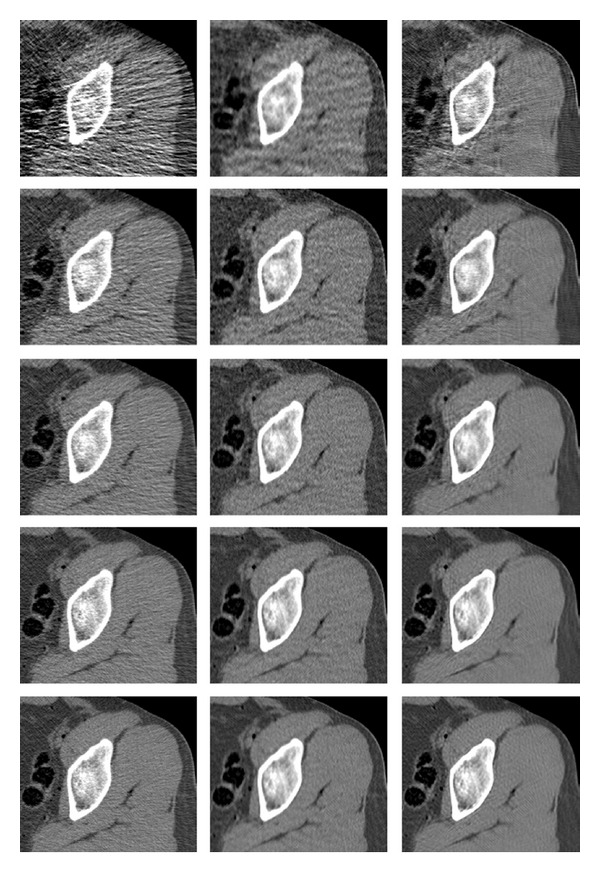
Columns, left to right: reconstruction by FBP, PWLS, and stage-II of our method. Rows, upper to lower: increasing signal energy corresponding to exposure levels of *λ*_0_ = [3 · 10^4^, 9.5 · 10^4^, 3 · 10^5^, 9.5 · 10^5^, 3 · 10^6^].

 The impact of noise level on reconstruction quality is demonstrated via an array of images in Figure 15 with FBP, PWLS, and our method.  Table 2 contains the standard quality measures—MSE and SSIM values for each noise level. The X-ray dose is increased exponentially from *λ*_0_ = 3 · 10^4^ to *λ*_0_ = 3 · 10^6^ (first to last rows in the figure), which results in linear improvement of the visual perception. The parameters of FBP were adjusted for each noise level; the parameters of the PWLS and our method are tuned for the exposure level of *λ*_0_ = 1.5 · 10^5^. This is the noise level used to simulate the training set in our algorithm.

In the strongest noise (row 1), our approach exhibits more streaks than the PWLS version since it is an FBP-based method (because our methods was not trained for this level of noise). In other cases the visual impression is similar for both algorithms. As the X-ray intensity increased, the quality of images produced by our method rises promptly to attain a nearly perfect image at the highest exposure. This testifies for the robustness of the training procedure with respect to noise level since the reconstruction results are adequate for X-ray doses which are either significantly higher or lower than the dose in the training set. Notice that the parameters of the PWLS algorithm are not optimal for the lowest noise level, where an oversmoothing is observed. The SNR values of PWLS images lead the charts at very low and very high exposure levels, while in rows 2, 3 of the table our method shows superior results. The SSIM measure implies that the performance of PWLS is very close to that of our method. FBP is comparable to these two algorithms at high X-ray intensity and is way below in the low-dose scenario. 

### 6.6. Aiming for Lowest MSE

**Figure 16:**
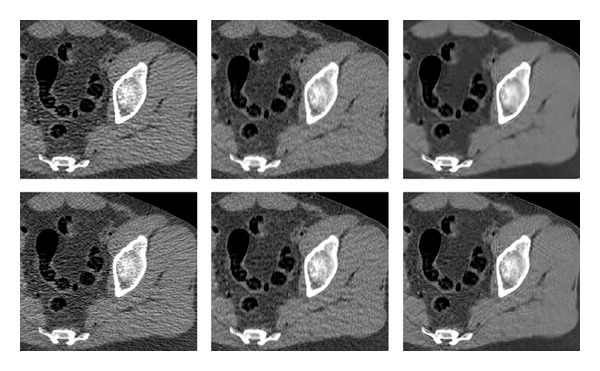
Upper row, left to right: reconstructions optimized for SNR values—FBP (28.5 dB), PWLS (30.6 dB), our method (31.41 dB). Lower row: reconstruction with higher spatial resolution—FBP (26.5 dB), PWLS (29.6 dB), our method (30.73 dB).

 Now we return to the question of whether MSE is an error measure relevant to radiological images. The images, presented earlier in Figure 7, were obtained by tuning the algorithms for high spatial resolution, at the price of noise level and the SNR value. Here we display three algorithms—FBP, PWLS, and our method—with parameters optimized for maximal SNR (we were not able to control the ATM filter behavior that way). With FBP, it is achieved by tuning the cut-off frequency of its sinogram filter; for PWLS, we have increased the weight of the Huber penalty component. Our method is manipulated by tuning the weight *μ* of the gradient-based component in (24), (26). In the upper row of Figure 16, reconstruction of the test image by those three methods with SNR-optimized parameters is displayed. The lower row contains the image versions from Figure 7; they were produced with parameters optimized for visual perception. Specifically, the spatial resolution was improved at expense of a tolerable additional noise. This display is given to show the tradeoff between the noise reduction and spatial resolution and visualize our stimulus in pursuing the latter virtue rather than the former.

### 6.7. Detecting Lesions in Noisy Images

**Figure 17:**
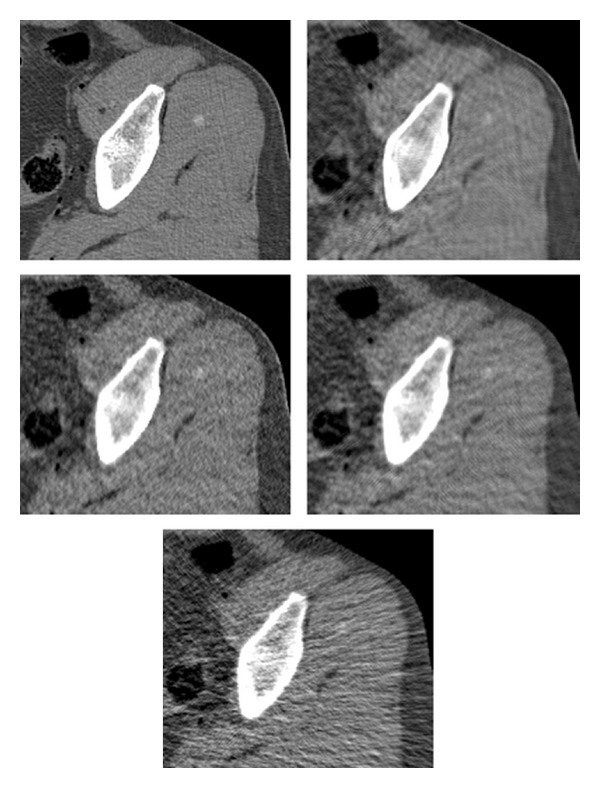
Detectability test for a small inserted lesion. Upper to lower rows, right to left: reference image, our method, PWLS, ATM, and FBP images. The HU window is [−220,350].

 A lesion detectability experiment is designed as follows: we add a small disk-shaped blob in the homogeneous region of test image. The average intensity of the lesion is 105 HU on the background tissue of average 54 HU. The experiment is conducted in conditions of strong noise, concealing the lesion spot in the FBP reconstruction. Explicitly, it corresponds to *λ*_0_ = 7 · 10^4^ photons. In Figure 17 the reconstruction of a region, containing the lesion, is displayed. We compare our algorithm with the PWLS, ATM, and the FBP. The parameters of the learned shrinkage were trained offline on the training data with the same noise energy; the PWLS and ATM were used with the same parameters as earlier for the following reasons. For PWLS, a manual tuning of the smoothing weight did not result in any improvement of visibility. For ATM, there is no intuitive way to change the four parameters for a higher noise level. The FBP was used with same cut-off frequency but higher order of the Butterworth window, which made the lesion more observable.

**Figure 18:**
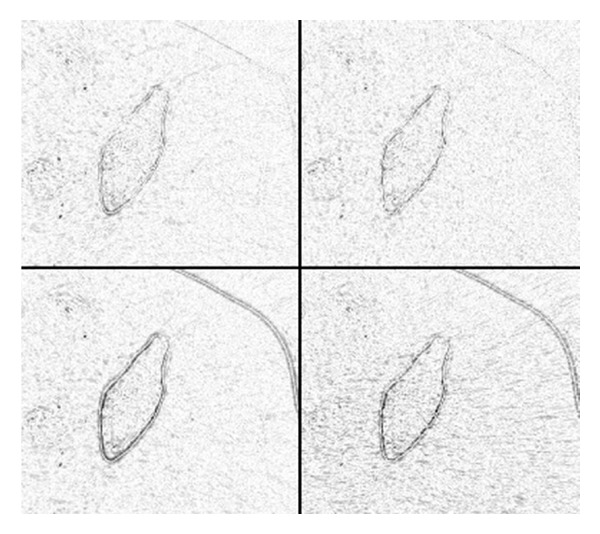
Error maps for the lesion detectability test, built with respect to the reference image. Upper to lower rows, left to right: our method, PWLS, ATM, and FBP images. Darker shade corresponds to higher error. The lesion is not observed in any version, which means it is recovered correctly by all the methods; however, in FBP image the lesion is obscured by streaks.

One can observe that the synthetic lesion (no similar structure was present in the training data) is recovered correctly and, in contrast with the FBP image, it can be clearly observed. PWLS produces an image which is more sharp and noisy than our result, and the ATM image is of a lower quality. The error images in Figure 18 (difference between the reconstruction and the reference) imply that the noise in the image produced by our method is lower than that in the PWLS reconstruction (this experiment is also the chance to compare the algorithms at a stronger noise). The lesion is not observed in any error image which means it is not lost in reconstruction; the problem with FBP is not that it fails to recover the lesion but the high streak-shaped noise obscuring it.

### 6.8. Design Parameters of the Proposed Method

**Figure 19:**
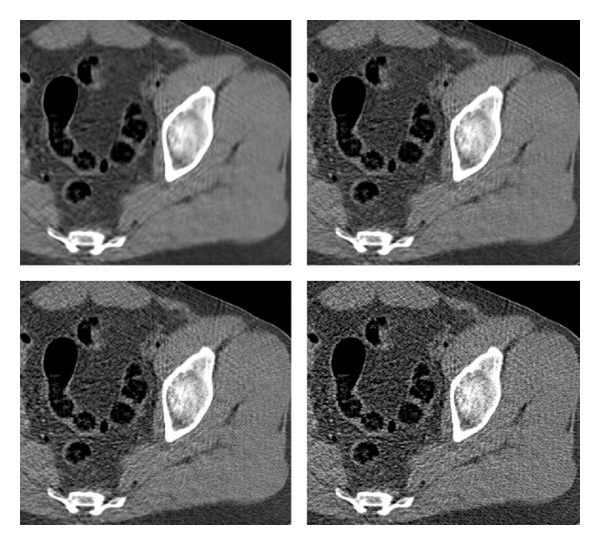
Reconstruction with learned shrinkage trained with the different values of the weight *μ*. Upper to lower rows, left to right: images corresponding to *μ* = [14.3,105.6,205.5,400].

 We now study the impact of various parameters appearing in the reconstruction chain. First we consider the objective function in (24). The weight *μ* controls the influence of the gradient-based component; when set to zero, the training leads to best MSE reduction. The influence of this component on the visual appearance of the image is observed in Figure 19. The presented sequence of images corresponds to values (these values are a subset of an exponential sequence of the *μ* range, swept in a numerical experiment. We chose these four values because they provide visibly different reconstructions.) *μ* = [14.3,105.6,205.5,400]. As *μ* grows, the blur, introduced by the reconstruction, is reduced; however, new artifacts arise. They result from strong influence of the gradient-based component. After a finer tuning we chose to use *μ* = 100, which produces the most visually appealing image. Its sharpness is near best, and artifacts are on the level of background noise. For real-life application, a more elaborate study by a radiologist may be needed to tune this parameter for clinical needs.

**Figure 20:**
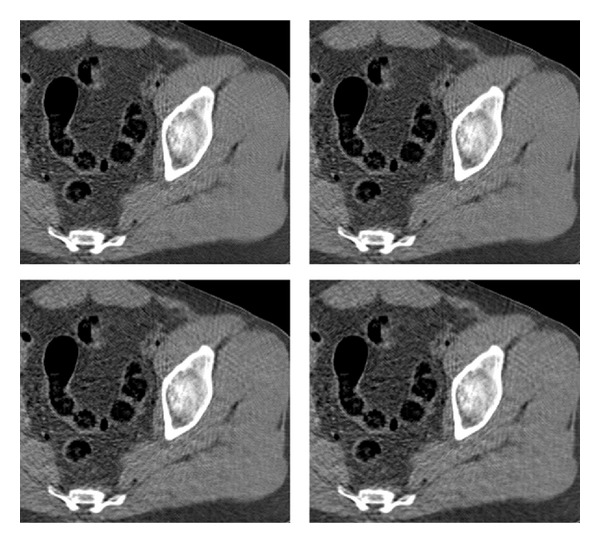
Left to right: reconstruction with learned shrinkage trained using varying regularization weight, *γ*_*I*_ = [0.001,0.0032,0.0316,0.1].

Another aspect of the training process is the regularization weights *γ* in (24) and *γ*_*I*_ in (26), which restrict the deviation of the shrinkage functions from identity. In general, using such regularization is a good practice to increase the robustness of the method by preventing the overfitting. Also, this helps reducing the impact of outlier examples on the shape of shrinkage functions. In our experiments, the influence of those regularization terms was not significant: when the weights *γ*, *γ*_*I*_ are decreased, the effect of learned shrinkage becomes stronger but no negative phenomena appear. This is observed in Figure 20, where a number of versions of a test image, associated with different values of *γ*_*I*_, are shown.

### 6.9. Local Impulse Response

**Figure 21:**
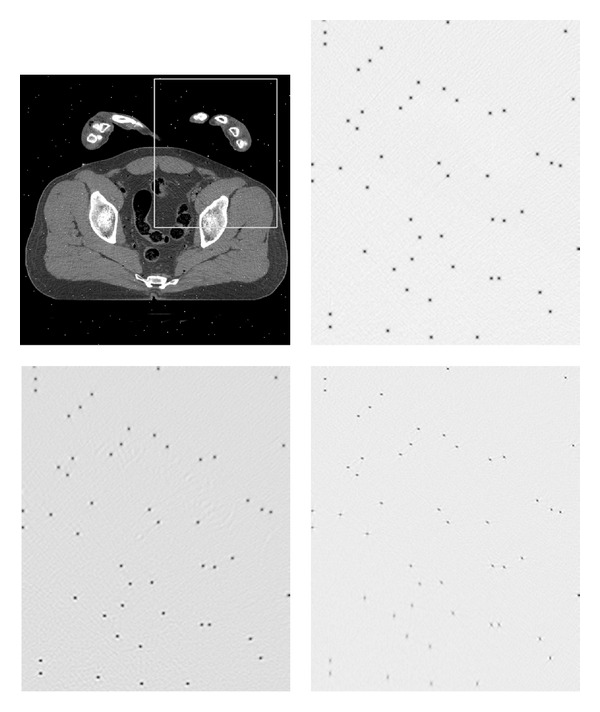
Upper left: reference image with added spikes. The LIR maps are shown in the marked region. Upper right: LIRs obtained with FBP reconstruction. Lower row, left to right: LIR maps obtained with our algorithm and the PWLS.

 The evaluation of spatial resolution in the CT images is carried out by computing the local impulse responses (LIR) of the projection-reconstruction operator in the image domain. The reference image is projected twice, one time in its original form and another with a random set of 212 single-pixel implants, scattered randomly in the image. The intensity of the implants is set to the maximal value present in the image. In Figure 21 we display an example region in a test image with added spikes and the corresponding maps of LIRs obtained by subtracting the two reconstructions—with and without spikes—by the compared methods. All the parameters of the compared methods are set as in the very first experiment. For each method, the full-width half-maximum (FWHM) measure is computed in all the locations and averaged. It is defined as follows: first, a 2-D image patch containing the response spot is resized into a ×16 larger image in order to reduce the discretization effect. Then, the number of pixels, which intensity is higher than half of the maximal value in the patch, is counted and divided by the refinement factor of 16.

**Figure 22:**
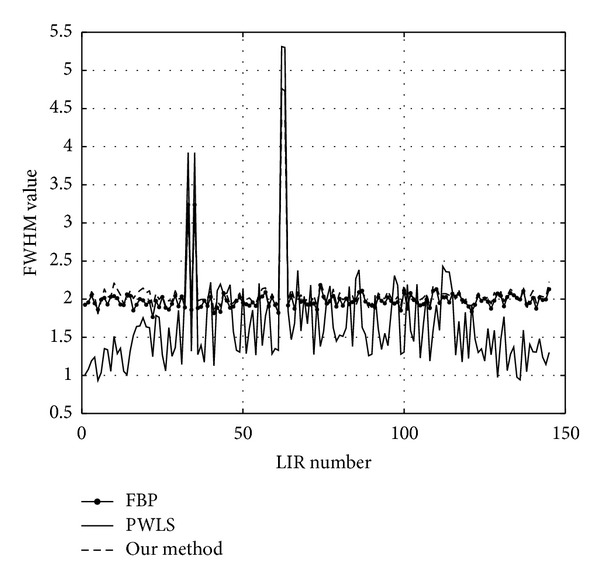
FWHM values in 212 random image locations.

The computation was done for FBP, PLWS, and our algorithm. An average FWHM value produced by stage I of our method is 2.11 pixels; the resolution is slightly improved to 2.06 pixels by the stage II. Notice that this postprocessing step simultaneously reduces the noise and increases the image sharpness; this virtue is attributed to the design of our error measure. The FBP exhibits the same average FWHM value—2.04 pixels. In Figure 21 both FBP and our method are seen to produce disk-shaped LIRs without distortions everywhere in the image. The situation is different with the data-adaptive PWLS, which achieves lower FWHM values—1.61 pixels on average—but displays an anisotropic smearing of the spikes. The graphs of FWHM values for all the LIRs are given in the Figure 22. 

### 6.10. Effective X-Ray Dose Reduction

 The reduction of noise and artifacts enables, effectively, reduction of the X-ray dose while keeping the level of image quality. We estimate the reduction factor by comparing the SNR and MSEg values of the reconstructed image, acquired with different X-ray doses (controlled by the source intensity *λ*_0_). The comparison is conducted between the standard FBP and the proposed reconstruction method.

**Figure 23:**
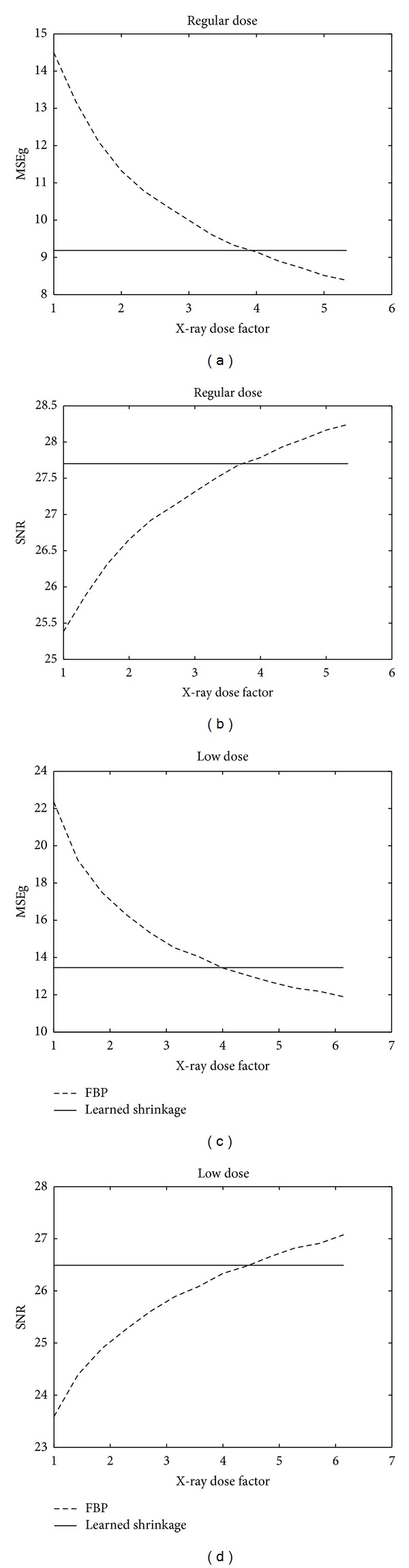
Effective dose reduction by the proposed method, with respect to the optimally tuned FBP algorithm. (a), (b) Regular X-ray dose. (c), (d) low X-ray dose. (a), (c) MSEg measure. (b), (d): SNR measure.

For each dose level, we tune the FBP parameters to choose a reconstruction with minimal MSEg value. In a second experiment, for each noise level, the FBP is tuned to maximize the SNR value. In Figure 23 we present the resulting comparison of these two measures for a low dose and a regular dose scan. The *x*-axis is scaled to show the dose reduction factor, while the MSEg or SNR values change along the *y*-axis. In the SNR graph we plot the values achieved by SNR-optimized FBP, and, in MSEg graph, the performance of MSEg-optimized FBP is displayed. Those are compared to the single SNR/MSEg value, achieved by our algorithm in the noise level it was trained for; the *x*-axis of the graphs was scaled to display the ratio between the X-ray dose levels for FBP and our method. All the four graphs point to the effective dose savings of factor ~4 when switching from the optimally tuned FBP to our method. 

## 7. Discussion and Conclusions

We have introduced a practical CT reconstruction algorithm which performs a non linear processing of the measurements and the reconstructed image. Both actions are aimed at high-quality reconstruction from data corrupted with shot noise and electronic noise. The defining parameters of the learned shrinkage are trained in an offline session on a set of available reference images. When applied to deteriorated measurements of new images, the algorithm produces a reconstruction which improves upon the standard FBP output and the nonlinear ATM filter and is comparable to the iterative PWLS reconstruction.

The learned shrinkage in the transform domain is a nonlinear two-dimensional filter applied in the domain of the noisy measurements. It is shown to be capable of substantially reducing the streak artifacts caused by the measurements' noise. Further postprocessing action is essentially a classical image denoising task, which is carried out without a comprehensive noise model and is aimed to minimize a specific error measure. It is also performed with the learned shrinkage. Our observations, supported by quantitative measures, point to the quality improvement this technique brings to the reconstructed images.

We remark that a potentially greater quality improvement would be achieved by exploiting data correlation in three dimensions (instead of processing 2D slices), similarl to three-dimensional adaptive filtering presented in [9]. Our algorithm naturally generalizes to the 3D setup—the extension would involve 3D discrete cosine transform and a quality measure computed in some volumes of the training data.

As with any algorithm, based on supervised learning, there is a concern of whether the medical anomalies and special objects will be faithfully recovered. In our simulations, the visual comparison of the images reconstructed with our method to the reference images confirms that the content is faithfully recovered. Also, we point to the fact that the processing is performed locally (11 × 11 squares) and in a transform domain; the action of the shrinkage operation is of statistical rather than geometric nature; thus it is improbable and it will be biased by specific anatomical structures. Still, only the practical realization of the algorithm and verification by clinical radiologists can resolve this concern.

The proposed algorithm requires no hardware changes in a working CT scanner and can be easily incorporated into the reconstruction software of one; thus, in practice it can be implemented in existing clinical machinery with small effort.

## Figures and Tables

**Table 1:** Quantitative measures for the compared algorithms.

Method	FBP	ATM	PWLS	Shrinkage
Thighs				
MSEg =				
MSE + grad	16.12 + 11.64	11.81 + 12.54	7.98 + 5.31	6.99 + 11.17

MSEg total	27.76	14.35	13.29	18.16

SNR (dB)	26.56	28.70	29.56	30.73
SSIM	0.8643	0.9124	0.9219	0.9398

Abdomen				
MSEg =				
MSE + grad	14.96 + 6.88	11.78 + 6.73	7.12 + 7.49	6.80 + 7.18

MSEg total	21.83	18.52	14.61	13.99

SNR (dB)	26.42	27.34	29.53	30.41
SSIM	0.8832	0.8985	0.9326	0.9483

Head				
MSEg =				
MSE + grad	6.99 + 11.40	6.99 + 11.39	3.31 + 7.99	2.77 + 6.38

MSEg total	18.40	18.38	11.30	9.15

SNR (dB)	30.84	30.84	31.94	33.74
SSIM	0.9764	0.9764	0.9783	0.9864

**Table 2:** Quantitative measures corresponding to the image array in Figure 15. The SNR is measured in HU window of [−220,350].

Noise level *λ*_0_	FBP		PWLS		Shrinkage	
	SNR	SSIM	SNR	SSIM	SNR	SSIM
3 · 10^4^	21.87	0.75	27.82	0.90	25.39	0.86
9.5 · 10^4^	26.53	0.86	28.97	0.91	29.88	0.92
3 · 10^5^	28.86	0.91	30.59	0.93	31.65	0.94
9.5 · 10^5^	30.67	0.93	32.62	0.95	32.43	0.95
3 · 10^6^	32.29	0.95	33.93	0.96	32.85	0.96
